# Surface Roughness and Microbial Adhesion on Four Provisional Prosthodontic Restorative Materials

**DOI:** 10.3390/dj13110498

**Published:** 2025-10-27

**Authors:** Ola Al Hatem, Joe C. Ontiveros, Donald M. Belles, Maria D. Gonzalez, Ransome van der Hoeven

**Affiliations:** 1 Department of Restorative Dentistry and Prosthodontics, School of Dentistry, The University of Texas Health Science Center, Houston, TX 77054, USA; olaalhatem@gmail.com (O.A.H.); joe.c.ontiveros@uth.tmc.edu (J.C.O.); donald.m.belles@uth.tmc.edu (D.M.B.); maria.d.gonzalez@uth.tmc.edu (M.D.G.); 2 Department of Diagnostic and Biomedical Sciences, School of Dentistry, The University of Texas Health Science Center, Houston, TX 77054, USA; 3 Iowa Institute for Oral Health Research, College of Dentistry, University of Iowa, Iowa City, IA 52242, USA; 4 Department of Periodontics, College of Dentistry, University of Iowa, Iowa City, IA 52242, USA

**Keywords:** poly (methyl methacrylate), dimethacrylate, zirconium oxide

## Abstract

**Objective:** The aim of this study was to evaluate surface roughness (R_a_) and microbial adhesion on four provisional prosthodontic materials in comparison to zirconium oxide. **Methods:** Four provisional prosthodontic restorative materials were evaluated in this study: poly methyl methacrylate (PMMA) acrylic resin (ALIKE; GC America Inc., Alsip, IL, USA), dimethacrylate (Bis-acryl) resin (Integrity; Dentsply Sirona, Charlotte, NC, USA), 3D-printed temporary crown and bridge resin (Formlabs Inc., Somerville, MA, USA), prepolymerized poly methyl methacrylate (milled PMMA) (Harvest Dental Laboratory Products, Brea, CA, USA), and zirconium oxide (Ivoclar Vivadent AG, Liechtenstein, Germany). A total of 90 samples were prepared and divided into two groups per material (treated and untreated). Provisional material samples were prepared per manufacturer’s instructions, polished with the same sequence using acrylic burs followed by Acrylipro silicone polishers (Brasseler, Savannah, GA, USA), and pumice with a goat brush. Zirconia was polished with a green grinding stone (ZR Grinders; Brassseler, Savannah, GA, USA), followed by a feather lite (Dialite ZR polisher; Brasseler, Savannah, GA, USA). The Ra of all samples was measured using a digital profilometer. Sterilized samples were incubated in Todd Hewitt yeast extract (THY) broth containing *Candida albicans* SC5314 and *Streptococcus mutans* BM71 at 37 °C under anaerobic conditions for 72 h. Subsequently, the number of colony-forming units (CFU) adhered to each sample was determined by serial dilution plating. Normality and homoscedasticity were assessed prior to statistical analysis. Welch’s ANOVA was then performed to evaluate differences among all samples, followed by Games–Howell post hoc tests for pairwise comparisons. A *p* < 0.05 was considered significant in all experiments. **Results:** Zirconia demonstrated the lowest surface roughness and significantly reduced adhesion of *S. mutans* and *C. albicans* compared to all other materials (*p* < 0.001). Milled PMMA exhibited significantly lower roughness and microbial adhesion than conventional PMMA (*p* < 0.001), with no significant difference from Printed PMMA in microbial adhesion. Additional pairwise differences were observed between Bis-acryl and PMMA (*p* = 0.0425), Milled and Printed PMMA (*p* < 0.0001), and Bis-acryl and Printed PMMA (*p* < 0.0001). **Conclusions:** Zirconia and milled PMMA showed superior surface properties and reduced microbial adhesion, supporting their use in long-term provisional restorations. Materials with higher microbial retention, such as self-curing PMMA, bis-acryl, and 3D-printed resins, may be less suitable for extended use. These findings guide material selection to improve clinical outcomes and highlight the need for further in vivo research.

## 1. Introduction

Advancements in dental materials and digital technologies have significantly improved restorative outcomes, clinical workflows, and patient satisfaction. However, the biological implications of these materials, particularly their interaction with oral microbiota, must be carefully evaluated prior to clinical implementation [1]. The attachment of certain micro-organisms to specific surfaces in the oral cavity and the subsequent formation of dental plaque on teeth and dental materials are primary causes of oral diseases such as denture stomatitis, candidiasis, gingival inflammation, peri-implantitis, and secondary caries [2,3,4].

Provisional prosthodontic restorations are essential in fixed and implant prosthodontics, serving to protect pulpal tissues, support soft tissue healing, maintain tooth position, and facilitate communication among clinicians, patients, and technicians [5,6,7]. There are several types of conventional prosthodontic provisional restorative materials that can be classified by the type of resin. They can be fabricated chairside and are used for direct and indirect restorations [2,8,9]. Regardless of the type of provisional prosthetic restoration material used, the length of time for which it remains intraorally before permanent restorations are inserted is an important factor in determining the health of the supporting teeth and surrounding periodontal tissues [9].

Poly (methyl methacrylate) (PMMA) has long been used for provisional restorations due to its affordability, biocompatibility, and ease of fabrication. However, it presents several limitations, including shrinkage, thermal damage, porosity, poor marginal fit, water absorption, and color instability [10]. Additionally, PMMA cannot be directly integrated into digital workflows. To address these issues, bis-acryl composite resins were developed. Made of an organic matrix and inorganic fillers, they offer improved handling, reduced shrinkage, lower tissue toxicity, better marginal adaptation, and minimal heat generation [8].

As materials have advanced, so have fabrication techniques. CAD/CAM technology now plays a central role in restorative dentistry, using subtractive (milling) and additive (3D printing) methods. Since its introduction in the 1980s, 3D printing has become increasingly accessible, with systems like SLA, SLS, and DLP [11]. Within this digital workflow, milled restorations from pre-polymerized acrylic blocks show superior color stability and improved marginal accuracy than conventionally processed resin [12]. Meanwhile, 3D-printed restorations offer comparable fit and patient satisfaction at lower cost, though further research is needed to evaluate their long-term clinical performance [7].

Despite these technological advances, microbial adhesion remains a critical concern. The oral cavity harbors diverse microbial species, including *Streptococcus mutans* and *Candida albicans*, which are associated with caries and denture stomatitis, respectively. Even in asymptomatic individuals, these organisms are frequently isolated from denture surfaces [13,14,15,16]. Surface roughness is a key determinant of microbial colonization. Bacteria preferentially adhere to rough surfaces, which offer protection from shear forces and facilitate biofilm formation [13,17]. A threshold roughness value (R_a_) of 0.2 μm has been established, above which plaque accumulation significantly increases [4,18]. The proliferation of initially adhered micro-organisms contributes substantially to early biofilm development [19].

Recent studies have shown that 3D-printed resins exhibit higher *C. albicans* adhesion compared to milled or conventionally fabricated materials [20]. Print orientation and surface roughness are critical factors; rougher surfaces and build angles such as 90° are associated with increased microbial colonization. Biofilm formation by oral Streptococci, Staphylococci, and Candida species is more pronounced on unpolished or poorly post-processed 3D-printed surfaces [21]. In contrast, CAD/CAM-milled materials, particularly polished PMMA, demonstrate lower microbial adhesion, making them preferable for high-risk patients [7].

This in vitro study aims to evaluate the microbial adhesion of *S. mutans* and *C. albicans* and the surface roughness of commonly used provisional restorative materials, auto-polymerizing PMMA, bis-acryl composite resin, 3D-printed resin, and milled PMMA, under standardized polishing conditions. Zirconium oxide ceramic is included as a control due to its consistently low surface roughness and minimal microbial colonization. The null hypothesis is that there are no statistically significant differences in surface roughness or microbial adhesion among the tested provisional materials.

## 2. Materials and Methods

### 2.1. Materials Used in This Study

A total of 90 experimental samples were fabricated using four provisional prosthodontic restorative materials and zirconia, as described in Table 1 (n = 18 per material). The materials included polymethyl methacrylate (PMMA) acrylic resin (ALIKE; GC America Inc., Alsip, IL, USA), dimethacrylate (Bis-acryl) resin (Integrity (IG); Dentsply Sirona, Charlotte, NC, USA), 3D-printed temporary crown and bridge resin (Formlabs Inc.), and prepolymerized milled PMMA (Harvest Dental Laboratory Products, Brea, CA, USA). Zirconium oxide (Ivoclar Vivadent AG, Liechtenstein, Germany) was used as the control group. The sample size was determined based on a pilot study and was considered sufficient to detect statistically significant differences using one-way ANOVA and post hoc comparisons with an alpha level of 0.05.

### 2.2. Sample Preparation

The 3D-printed temporary crown and bridge materials (Formlabs Inc.) were first prepared as follows: An STL (Standard Tessellation Language) file of the samples was designed using 3D modeling software (Meshmixer; Autodesk Inc., San Rafael, CA, USA). The samples were printed in 10 × 5 × 3 mm dimensions and at a 50-micron layer line resolution using an in-office stereolithography 3D printer (Form 3B+; Formlabs, Somerville, MA, USA) oriented at 90 degrees on a stainless-steel build platform. Printed samples were washed in Formwash in 99% alcohol for 3 min and dried until a white powdery coat appeared, according to the manufacturer’s instructions. Subsequently, the samples were removed from the build platform by wedging with a sample removal tool. Post-curing was conducted in two steps: The first was 60 °C for 20 min after drying of the samples. The second was after removing supports with a disk and sandblasting to remove the powdery coat. To ensure uniform sample dimensions, a silicone rubber mold (15A Silicone Mold Making All-In-One Kit, Let’s Resin, Shenzhen Yi You Life Technology Co., Ltd., Shenzhen, China) making kit was used by mixing 1:1 ratios of part A and part B. The previously printed samples were arranged in rows in a container, and the silicone mix was poured. The setting time for silicone was 12 h at room temperature. Following the 12-h setting time, the next two sets of samples, the PMMA (ALIKE; GC America Inc., Alsip, IL, USA) and Bis-acryl Integrity Multi-Cure (IG; Dentsply Sirona, Charlotte, NC, USA), were fabricated. The PMMA was prepared following the manufacturer’s instructions by mixing one part liquid and three parts powder by volume in a mixing cup for 15 s, thereafter inserting it immediately into the silicone molds for a working time of 30–45 s, and leaving it for 2 min. To accelerate the curing time, samples were immersed in warm water at approximately 44 °C, following the manufacturer’s instructions. Bis-acryl was dispensed, using self-mixer tips, into the same molds, allowed to set for 2–4 min after the start of mixing, and covered with a glass slab; thereafter, it was light-cured with a halogen light for 20 s on each surface to achieve definitive hardness. The oxygen inhibition layer was then removed with alcohol wipes. For the last provisional material category, milled PMMA, the samples were milled from a prepolymerized disk (Temp Esthetic 98 PMMA; Harvest Dental Laboratory Products, Brea, CA, USA) by a five-axis mill using the same STL file. For the control group, the samples were milled from a prepolymerized PMMA disk (Temp Esthetic 98 PMMA; Harvest Dental Laboratory Products, Brea, CA, USA) or a zirconium oxide disk (IPS e.max ZirCAD MT Multi; Ivoclar Vivadent AG, Germany), respectively, with a five-axis mill using the same STL file.

### 2.3. Polishing Sequence

The same polishing sequence for all four materials was used to standardize the final finish. Starting with acrylic burs to remove supports and smooth touchpoint areas, followed by Acrylipro silicone acrylic polishers (Brassseler, Savannah, GA, USA) in the recommended sequence steps of green coarse, blue medium, and yellow fine. Finally, a goat hairbrush with medium and fine pumice was used. Each one of the steps was conducted for 10 s on each surface. Zirconia was polished with a green grinding stone (ZR Grinders; Brassseler, Savannah, GA, USA), followed by Featherlite (Dialite ZR polisher; Brassseler, Savannah, GA, USA) [22]. All provisional and zirconia samples were then placed in a bag of distilled water for 10 min in an ultrasonic bath (Cole-Parmer; Thermo Fisher Scientific, Waltham, MA, USA). Provisional materials were maintained in a humidified storage container at ambient temperature, whereas zirconia specimens were stored under dry conditions at the same temperature.

### 2.4. R_a_ Measurements

To measure R_a_, a pre-calibrated digital profilometer (Digiprofilo I; Digiwork Instruments, Concord, ON, USA) was used. The instrument consists of a general-purpose piezoelectric probe (SFP-2001) (Blatek Industries, Inc., Boalsburg, PA, USA) an R_a_ measurement range of 0.03 µm~6.3 µm/1 µm, a cut-off wavelength of 0.8mm, and a traverse length of 3.00 mm. Six total measurements were recorded for each of the test and control samples, as well as the zirconia control samples. The average of the six measurements per sample was used to calculate the mean average for each sample. The samples were fixated with a pair of cotton pliers during measurements. The digital profilometer was calibrated between samples to ensure consistent accuracy.

### 2.5. Microbial Adhesion Assay

Cultures of *C. albicans* SC5314 and *S. mutans* BM71 were grown overnight in Yeast Peptone Dextrose (YPD) and Todd–Hewitt Yeast extract (THY), respectively, at 37 °C on an orbital shaker (Barnstead MaxQ 4000 Digital Orbital Incubator Shaker, Marshall Scientific, Hampton, NH, USA) at 200 rpm. Thereafter, the cultures were normalized to an Optical Density (OD) = 0.1 using an Eppendorf BioPhotometer (Eppendorf AG, Hamburg, Germany). All provisional and zirconia samples were sterilized on both sides using an ultraviolet light for a duration of 30 min. Using sterile forceps, the sterilized samples were placed into wells of a 12-well tissue culture plate containing normalized cultures of *C. albicans* and *S. mutans* in THY. The plates were incubated at 37 °C in an anaerobic chamber containing a GasPak (Becton, Dickinson and Company (BD), Franklin Lakes, NJ, USA) for 72 h. As a negative control, provisional and zirconia samples were incubated in the presence of THY for the same duration. Thereafter, the samples were washed three times in Phosphate-Buffered Saline (PBS) and subjected to sonication in an ultrasonic bath for two rounds of 30 s and subsequently vortexed for 30 s. Then, 10-fold serial dilutions of each sample were prepared, and the dilutions were plated on THY agar. The plates were incubated at 37 °C overnight, and the number of colonies was counted for each dilution using a light microscope; then, the number of colonies in each sample was determined. The experiment was repeated three times, with three samples tested per material.

### 2.6. Statistical Analysis

Prior to conducting statistical tests, data were assessed for the normality and homogeneity of variances using the Shapiro–Wilk and Levene’s tests, respectively. Welch’s ANOVA was conducted to determine statistical significance among all samples, followed by Games–Howell post hoc comparisons. A *p* < 0.05 was considered significant in all experiments. The statistical analysis for all experiments was performed using GraphPad Prism version 10.0 (GraphPad Software, San Diego, CA, USA) and IBM SPSS Statistics v31.3.

## 3. Results

**Figure 1:**
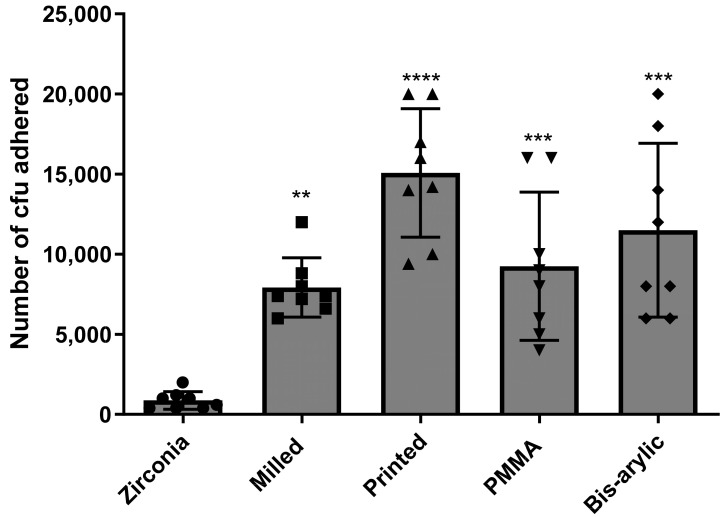
Combined mean number of CFUs of *C. albicans* and *S. mutans* that adhered to all four provisional materials and zirconia. A total of nine samples per group were tested, and colonies were counted via direct plate count and light microscopy. (**** *p* < 0.0001, *** *p* < 0.001 and ** *p* < 0.01).

Statistical analysis confirmed that the data for surface roughness (Table 2) and microbial adhesion of *S. mutans* and *C. albicans* were normally distributed across all materials, as indicated by the Shapiro–Wilk test (*p* > 0.05 for all groups). However, Levene’s test revealed significant differences in variances for both roughness (W = 2.5090, *p* = 0.0478) and adhesion (W = 4.8779, *p* = 0.0031), supporting the use of Welch’s ANOVA and Games–Howell post hoc comparisons. For roughness, Welch’s ANOVA indicated a significant difference among materials (F = 132.59, df = 4, 71), with Zirconia (0.39 ± 0.09) showing significantly lower roughness compared to PMMA (1.68 ± 0.23), Bis-acryl (1.61 ± 0.23), Milled, and Printed (1.25 ± 0.17) (*p* < 0.0001) (Table 3). Additional significant differences were observed between Bis-acryl vs. PMMA (*p* = 0.0425), Milled vs. PMMA (*p* < 0.0001), and Milled vs. Printed (*p* < 0.0001). For microbial adhesion, Welch’s ANOVA also revealed significant differences (F = 15.62, df = 4, 22), with Zirconia (875 ± 555) exhibiting significantly lower adhesion compared to Printed (15,075 ± 4017, *p* < 0.0001), PMMA (9250 ± 4621, *p* = 0.0008), Milled (7925 ± 1848, *p* = 0.0013), and Bis-acryl (11,500 ± 5425, *p* < 0.0001) (Figure 1). Further significant pairwise differences were found between Bis-acryl vs. Printed (*p* < 0.0001) and Milled vs. PMMA (*p* = 0.0011), while comparisons between Milled vs. Printed (*p* = 0.4703) and PMMA vs. Printed (*p* = 0.1130) were not statistically significant.

## 4. Discussion

The null hypothesis was rejected following the statistical analysis, confirming significant differences in surface roughness and microbial adhesion among the tested provisional restorative materials. This study assessed surface characteristics and microbial colonization by *S. mutans* and *C. albicans* on self-cure PMMA, CAD/CAM-milled PMMA, bis-acryl composite resin, and 3D-printed resin, with zirconia serving as the control. Significant differences in mean R_a_ values were observed, with zirconia exhibiting the smoothest surface and the lowest microbial adhesion. Among the provisional materials, milled PMMA demonstrated superior smoothness, whereas 3D-printed resin showed significantly greater microbial adhesion compared to milled PMMA. However, no statistically significant differences in microbial adhesion were found among the printed resin, bis-acryl, and self-cure PMMA groups.

The observed differences in microbial adhesion are primarily attributable to the intrinsic properties of each material, namely surface roughness, chemical composition, and fabrication method. Furthermore, in vivo polishing protocols are operator-dependent and often result in higher R_a_ values compared to standardized laboratory conditions. Among the materials studied, zirconia consistently exhibited the lowest levels of microbial colonization. This finding is consistent with its highly polished, dense ceramic surface and low surface free energy, which collectively contribute to its reduced microbial affinity. [23]. These characteristics reduce the availability of micro-retentive features and limit bacterial and fungal adhesion. Additionally, zirconia’s hydrophobic nature and absence of organic components further contribute to its resistance to biofilm formation.

Milled polymethyl methacrylate (PMMA) materials demonstrated significantly lower microbial adhesion compared to conventional PMMA and bis-acryl resins [11]. This reduction is primarily attributed to the enhanced surface smoothness and reduced porosity achieved through computer-aided design and computer-aided manufacturing (CAD/CAM) milling processes [24]. Additionally, the diminished adherence of *S. mutans* to PMMA surfaces may be influenced by the material’s higher surface energy and increased hydrophobicity, which collectively hinder bacterial colonization [9]. The controlled polymerization inherent to milled PMMA further contributes to its superior surface quality by minimizing defects and residual monomer content, thereby limiting microbial retention. Notably, residual methyl methacrylate monomers present in PMMA resins have also been implicated in affecting the viability of *S. mutans* cells [9].

In contrast, self-curing PMMA showed a significantly higher microbial adhesion. As a polymer-based material, PMMA possesses a relatively porous and heterogeneous surface that facilitates microbial entrapment. Its moderate hydrophilicity and susceptibility to water sorption and surface degradation over time increase surface roughness and promote biofilm development, an important consideration for long-term provisional applications where microbial control is critical [25,26]. The high surface roughness of the conventional group can be attributed to the air bubbles incorporated through hand mixing of liquid and powder during filling of the external mold [2,7].

Bis-acryl composite resins also demonstrated elevated microbial adhesion. Their formulation includes multifunctional methacrylate monomers and inorganic fillers, which contribute to a rougher surface texture and microstructural irregularities [27]. These features provide niches for microbial attachment, and the presence of residual monomers may further enhance colonization, especially under intraoral conditions where mechanical and chemical stresses are prevalent.

Similarly, printed resin materials exhibited relatively high microbial adhesion, comparable to conventional PMMA. The additive manufacturing process introduces layer lines and micro-roughness, which serve as retention sites for microorganisms [2]. Moreover, variability in polymerization and the surface energy of photopolymer resins used in 3D printing may influence microbial behavior [28]. Despite technological advancements, the surface refinement of printed resins remains inferior to that of milled or ceramic materials [7,22].

This study has several limitations that should be considered when interpreting the findings. First, the in vitro conditions used do not fully replicate the complexity of the oral environment, where factors such as salivary flow, temperature fluctuations, mechanical forces, and host immune responses can significantly influence microbial behavior and material degradation. Second, microbial adhesion was assessed using cultures of *S. mutans* and *C. albicans*, which does not reflect the multispecies nature of oral biofilms. In clinical settings, microbial communities interact synergistically and competitively, affecting colonization dynamics and pathogenicity. Third, only one commercial brand per material category was evaluated, limiting the generalizability of the results. Variations in chemical composition, filler content, and manufacturing protocols across brands may lead to different surface characteristics and microbial responses. Lastly, surface roughness was measured using contact profilometry, which may introduce variability due to stylus geometry and potential surface disruption. More precise non-contact methods, such as optical or atomic force profilometry, could enhance measurement accuracy in future studies.

Future research should expand the range of brands and compositions studied, incorporate non-contact profilometry for more accurate surface measurements, and simulate intraoral conditions to better understand clinical performance. Investigating the impact of polishing protocols and post-processing treatments on microbial adhesion could also help refine material selection for provisional restorations.

## 5. Conclusions

This study identified significant differences in surface roughness and microbial adhesion among provisional restorative materials. Zirconia and milled PMMA demonstrated superior performances, with milled PMMA emerging as the more clinically favorable option due to its smooth surface and reduced microbial colonization. In contrast, self-curing PMMA and bis-acryl resins showed higher microbial adhesion, limiting their suitability for extended use. Although 3D-printed resins offer design flexibility, their surface roughness and elevated microbial retention present challenges for long-term clinical application. To translate these findings into clinical practice, further in vivo studies are essential to explore the impact of polishing protocols, post-processing treatments, and brand variability on microbial adhesion and surface integrity. For long-term provisional restorations, clinicians should favor milled PMMA or zirconia, particularly in patients requiring optimal plaque control and soft tissue health.

## Figures and Tables

**Table 1:** Description of materials used in this study.

Polymerization Method	Material	Type	Composition and Properties	Manufacturer
3D Printing	Formlabs Temp C&B	Liquid photopolymers	Methacrylate-based resin with high filler content; flexural strength ≥ 100 MPa; density 1.4–1.5 g/cm^3^; viscosity 2500–6000 MPa·s	Formlabs Inc., Somerville, MA, USA
CAD/CAM Resin Disk Milling	Harvest Dental Resin Disk	Prepolymerized PMMA	Poly(methyl methacrylate) [CAS 9011-14-7], titanium dioxide [CAS 1317-80-2], iron oxide [CAS 1309-37-1], fluorescent pigments (e.g., calcium, strontium, magnesium sulfides), EDMA crosslinker	Hersteller, Prinsessegracht 20, The Hague, Netherlands
Self-cure Powder/Liquid Mix	Alike	PMMA	Powder: PMMA polymer; Liquid: methyl methacrylate monomer; self-curing via free radical polymerization; minimal shrinkage; low water absorption	GC America (COE), Alsip, IL, USA
Automixing Gun with Dispensing Tips	Integrity Multi·Cure	Dimethacrylate (Bis-acryl)	Bis-acryl composite resin with Bis-GMA, UDMA, TEGDMA; dual-cure (self + light); flexural strength ~850 MPa; added fluorescence; 10:1 automix ratio	Dentsply Caulk, Milford, DE, USA
CAD/CAM IPS e.max ZirCAD	IPS e.max ZirCAD MT Multi	Zirconium Oxide	Multi-layered zirconia: 5Y-TZP (translucent incisal zone) + 4Y-TZP (opaque dentin zone); flexural strength ~850 MPa; fracture toughness >5 MPa·m^½^; natural gradient of translucency	Ivoclar Vivadent AG, Liechtenstein, Germany

**Table 2:** Mean R_a_ values for provisional prosthodontic restorative materials and zirconia.

	Zirconia	Milled	Printed	PMMA	Bis-Acryl
Mean	0.39 (±0.09)	1.12 (±0.19)	1.25 (±0.17)	1.68 (±0.23)	1.61 (±0.23)

**Table 3:** Comparison of R_a_ between the provisional prosthodontic restorative materials and zirconia.

Games–Howell Comparisons Test	*p* Value	Summary
Zirconia vs. Milled	<0.0001	****
Zirconia vs. Printed	<0.0001	****
Zirconia vs. PMMA	<0.0001	****
Zirconia vs. Bis-acryl	<0.0001	****
Milled vs. Printed	0.022	*
Milled vs. PMMA	<0.0001	****
Milled vs. Bis-acryl	<0.0001	****
Printed vs. PMMA	<0.0001	****
Printed vs. Bis-acryl	<0.0001	****
PMMA vs. Bis-acryl	0.8179	ns

Statistical significance: **** *p* < 0.0001, * *p* < 0.05, ns = not significant.

## Data Availability

The original contributions presented in the study are included in the article; further inquiries can be directed to the corresponding author.

## Abbreviations

The following abbreviations are used in this manuscript:

PMMA	Poly Methyl Methacrylate
SLA	Stereolithography
SLS	Selective Laser Sintering
DLP	Digital Light Processing
R_a_	Mean Surface Roughness
OD	Optical Density
THY	Todd Hewitt Yeast Extract
